# Prognostic Role of Initial Thromboelastography in Emergency Department Patients with Primary Postpartum Hemorrhage: Association with Massive Transfusion

**DOI:** 10.3390/jpm14040422

**Published:** 2024-04-16

**Authors:** Sang Min Kim, Chang Hwan Sohn, Hyojeong Kwon, Seung Mok Ryoo, Shin Ahn, Dong Woo Seo, Won Young Kim

**Affiliations:** Department of Emergency Medicine, University of Ulsan College of Medicine, Asan Medical Center, Seoul 05519, Republic of Korea; d200226@amc.seoul.kr (S.M.K.); hyojeong1214@amc.seoul.kr (H.K.); chrisryoo@amc.seoul.kr (S.M.R.); ans1023@amc.seoul.kr (S.A.); sdw@amc.seoul.kr (D.W.S.); d080593@amc.seoul.kr (W.Y.K.)

**Keywords:** postpartum hemorrhage, blood transfusion, thromboelastography

## Abstract

Background: The early prediction of the need for massive transfusions (MTs) and the preparation of blood products are essential for managing patients with primary postpartum hemorrhage (PPH). Thromboelastography (TEG) enables a thorough evaluation of coagulation status and is useful for guiding the treatment of hemorrhagic events in various diseases. We investigated the role of TEG in predicting the need for MT in patients with primary PPH. Methods: A retrospective observational study was conducted in the emergency department (ED) of a university-affiliated, tertiary referral center between November 2015 and August 2023. TEG was performed upon admission. We defined MT as the requirement for transfusion of more than 10 units of packed red blood cells within the first 24 h. The primary outcome was the need for MT. Results: Among the 184 patients with initial TEG, 34 (18.5%) required MT. Except for lysis after 30 min, the MT and non-MT groups had significantly different TEG values. Based on multivariate analysis, an angle < 60 was an independent predictor of MT (odds ratio (OR) 7.769; 95% confidence interval (CI), 2.736–22.062), along with lactate (OR, 1.674; 95% CI, 1.218–2.300) and shock index > 0.9 (OR, 4.638; 95% CI, 1.784–12.056). Alpha angle < 60 degrees indicated the need for MT with 73.5% sensitivity, 72.0% specificity, and 92.3% negative predictive value. Conclusions: Point-of-care testing of TEG has the potential to be a useful tool in accurately predicting the necessity for MT in ED patients with primary PPH at an early stage.

## 1. Introduction

Despite recent advancements in obstetric protocols for managing significant hemorrhage, postpartum hemorrhage (PPH) contributes a major cause of maternal mortality worldwide [1]. Although PPH is defined as a blood loss of more than 500 mL, healthy pregnant patients could endure a blood loss of 500–1000 mL without experiencing any symptoms or indication of hypovolemia [2,3]. Primary PPH is the condition referring to a significant loss of blood, either with symptoms or evidence of low blood volume, amounting to at least 1000 mL within 24 h following childbirth or delivery of the fetus [2,4]. Timely administration of blood transfusions is essential for reducing maternal mortality due to severe hemorrhage [5]. It is, therefore, crucial to improve outcomes by accurately predicting the need for massive transfusion (MT) and ensuring the availability of appropriate blood products in advance.

Objective parameters have been developed to predict the severity of bleeding and the need for MT because clinicians often provide inaccurate and underestimated estimates of blood loss [6]. The correlation between the reduction in hematocrit and the severity of blood loss is weak, and it is not clinically available in emergency conditions [6]. The shock index, which is the ratio of heart rate (HR) to systolic arterial blood pressure (BP), along with lactate, has been identified as a reliable indicator for predicting the need for MT in patients with PPH [7,8]. Furthermore, a recent statement on patient blood management for PPH recommends using either conventional coagulation tests, such as platelet count, prothrombin time (PT), activated partial thromboplastin time (aPTT), and fibrinogen, or viscoelastic hemostatic tests to guide the appropriate use of blood components [9].

Thromboelastography (TEG) is a point-of-care test that rapidly evaluates each phase of coagulation, from clot initiation to termination and fibrinolysis [10]. TEG has quicker turnaround times than conventional coagulation tests and physicians use TEG for guiding hemostatic therapy in cardiac surgery, transplantation surgery, and trauma [11,12,13]. Furthermore, TEG could be helpful to identify hemorrhagic events in acute stroke [14], and association with sepsis-induced coagulopathy has been studied before [15]. Moreover, previous studies have shown that utilizing TEG during the postpartum period results in a decrease in both the amount of bleeding and the need for transfusion [16,17]. However, data on the role of TEG in predicting the need for MT are limited in PPH.

We hypothesized that TEG can be a valuable tool in promptly evaluating the risk assessment of the need for MT upon admission to the emergency department (ED). We aimed to assess the use of TEG as an early predictor for the requirement of MT in A&E patients with PPH.

## 2. Materials and Methods

### 2.1. Study Design and Setting

We conducted a retrospective observational study in the ED of a university-affiliated tertiary hospital with 2800 beds in South Korea from November 2015 to August 2023. We included patients with primary PPH who visited the A&E department and underwent TEG upon admission. The definition of primary PPH was hemorrhage that required fluid resuscitation or transfusion within 24 h of delivery. Patients who were referred from obstetric clinics or other hospitals after delivery for the evaluation and management of PPH were also included. Patients who were not coagulable and did not provide initial TEG data were excluded. The requirement of MT was the primary outcome of this study. We defined MT as the transfusion of packed red blood cells of more than 10 units within the first 24 h of PPH [18]. To determine the MT, we assessed the total volume of transfused blood at both the prehospital level and the hospital level, including the A&E and general ward or the intensive care unit (ICU).

We divided patients into two groups: the non-MT group and the MT group, according to whether there was a requirement for MT or not. We compared several factors, including the clinical, baseline characteristics, and initial laboratory findings between two groups.

The Ethics Committee of our institution approved the study and allowed waiving the requirement for informed consent due to the study’s retrospective characteristics (IRB No. 2023-1392).

### 2.2. Data Collection

We extracted the clinical and baseline characteristics of patients from medical records, including age, type of delivery, parity, initial vital signs, initial mental status, initial laboratory findings, amount of blood transfusion, and clinical outcomes (length of hospital stay, ICU admission, embolization, hysterectomy, and in-hospital death). We assessed the patient’s mental status during the triage stage upon admission at the ED using the AVPU scale (Alert/Verbal/Painful/Unresponsive). We measured initial vital signs, including blood pressure (systolic and diastolic), pulse rate, and body temperature, upon admission. The initial shock index was determined by analyzing the initial vital signs. The ISTH DIC score was calculated using modified criteria associated with pregnancy, as previous research suggested, i.e., a pregnancy-modified ISTH DIC score ≥ 26 [19]. During the study period, the study institution established protocols for MT for patients with PPH in February 2022. Before the guideline was established, the decision about whether to perform a blood transfusion, as well as the type and amount of blood, was made by emergency physicians or obstetricians treating patients with primary PPH. Two ED physicians performed the data collection based on the pre-drafted clinical reporting form. One of the two ED physicians confirmed the fullness and preciseness of each data extraction form.

### 2.3. Thromboelastography

TEG was performed upon admission. Approximately 4 mL of whole blood was obtained in vials containing citrate along with the initial lab test. TEG analysis was performed utilizing a computerized coagulation analyzer (Model 5000; Haemonetics Corporation, Boston, MA, USA). The TEG analyzer evaluates the physical characteristics of the blood clot and documents the dynamic changes in the sample while the cups containing the whole blood samples rotate and oscillate. The recorded factors are the following: reaction time (R, measured in minutes, which is related to initial fibrin formation), kinetics (K, measured in minutes, time taken to achieve a certain level of clot strength dependent on fibrinogen), alpha angle (α, which measures the speed at which fibrin build up and cross-linking takes place, which is associated with the clot growth kinetics), maximum amplitude (MA, measured in mm, which represents the ultimate strength of the fibrin clot, dependent on platelets and fibrin), and lysis after 30 min (LY30, amplitude at 30 min, which reflects the percentage decrease in amplitude after MA, associated with fibrinolysis). We used TEG software (TEG Analytic Software 4.2.3; Haemonetics Corporation) for analysis, and all assays were conducted and evaluated according to the instructions provided by the manufacturer.

### 2.4. Statistical Analysis

Continuous variables that have normal distributions are presented as mean ± standard deviation. On the other hand, variables that have skewed distributions are presented using the median and interquartile range (IQR). Numbers and percentages are used to present categorical variables. We compared the variables between the non-MT group and the MT group using statistical tests that were appropriate for each variable type. The Wilcoxon rank-sum test or Student’s t-test were used for continuous variables, while Fisher’s exact test or the chi-square test were used for categorical variables. TEG analysis comprises the computation of the area under the receiver operating characteristic (ROC) curve, which is commonly referred to as AUC. We determined the cutoffs for TEG values, including K, Alpha angle, and MA, which represent an AUC of more than 0.750. We used methods based on ROC, specifically the Youden index, for this determination. We conducted multivariate logistic regression analysis to identify independent factors associated with the need for MT. We included variables that were found to be related to the need for MT in the univariate analysis in the logistic regression analysis. After confirming multicollinearity through linear regression, we conducted multivariate analysis using the backward stepwise regression process. The adequacy of the logistic model was assessed using the Hosmer–Lemeshow test. We presented the findings of the multivariate logistic regression analysis as odds ratios (OR) and 95% confidence intervals (CI). Furthermore, we determined the sensitivity, specificity, positive predictive value (PPV), and negative predictive value (NPV) using conventional statistical methods. The cutoff value for statistical significance was a two-sided *p* < 0.05. We performed the statistical analyses utilizing PASW Statistics for Windows Version 23.0 (IBM Corp. in Armonk, NY, USA).

## 3. Results

### 3.1. Baseline and Clinical Characteristics

**Figure 1:**
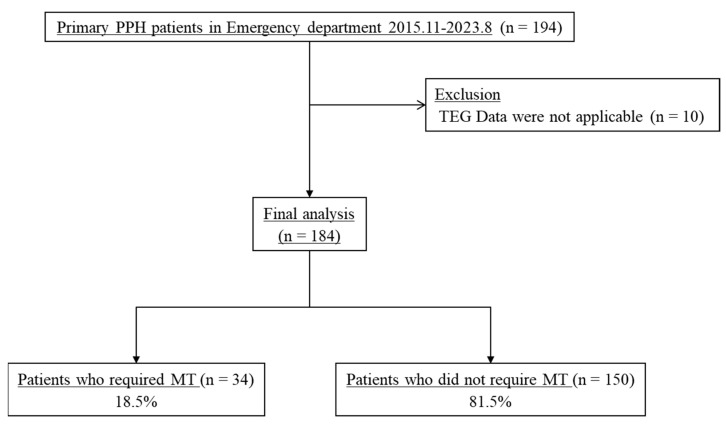
Patient flow diagram. ED, emergency department; PPH, postpartum hemorrhage; TEG, thromboelastography; MT, massive transfusion.

We identified a total of 194 patients with primary PPH during the study period. We excluded 10 patients from the analysis because their TEG data were not applicable, as the value of K and R were not provided due to extremely hypo coagulable state. Out of the 184 patients enrolled, 34 (18.5%) required MT (Figure 1).

Table 1 represents the clinical and baseline variables of patients categorized based on the need for MT. There were no significant differences in age, parity, or delivery type between the two groups. There was a significant difference in mental status between the two groups (*p* < 0.001). Patients in the MT group had significantly lower systolic (103.0 vs. 115.0), *p* = 0.001) and diastolic BP (63.0 vs. 73.0, *p* = 0.003), while pulse rate was significantly higher in the non-MT group (114.0 vs. 90.0, *p* < 0.001). There was no significant difference in the body temperature between the groups. The initial shock index was significantly higher in the MT group compared to the non-MT group (1.1 vs. 0.8, *p* < 0.001).

The initial laboratory findings, amount of blood transfusion, and clinical outcomes were compared according to the requirement for MT, as shown in Table 2. The initial lactate level was significantly higher in the MT group compared to the non-MT group (2.8 vs. 2.1, *p* = 0.006). Hemoglobin, hematocrit, platelet, and fibrinogen levels were significantly lower in the MT group. Otherwise, the PT (INR) was significantly higher in the MT group. The pregnancy-modified ISTH DIC score was significantly higher in the MT group than the non-MT group (51.0 vs. 26.0, *p* < 0.001). There was no significant difference in FDP level between the groups. The MT group received a significantly higher amount of packed red blood cell transfusion (12.0 vs. 3.0, *p* < 0.001). Fresh frozen plasma (11.0 vs. 2.0, *p* < 0.001) and platelet concentrates (10.0 vs. 0.0, *p* < 0.001) were given more to the MT group than the non-MT group. Embolization and hysterectomy were more commonly performed in the MT group. The length of hospital stay was shorter in the non-MT group. Only one patient who required MT died during her hospital stay.

### 3.2. Thromboelastographic Analysis

Table 3 shows the TEG parameters for each group. The MT group had higher levels of R and K compared to the non-MT group. Otherwise, the MT group had lower levels of alpha angle (47.5 vs. 68.0, *p* < 0.001) and MA (48.9 vs. 64.1, *p* < 0.001). There were no significant differences in LY30 between the two groups. The K has the highest discriminating power of 0.813 of AUC, followed by MA and alpha angle.

**Figure 2:**
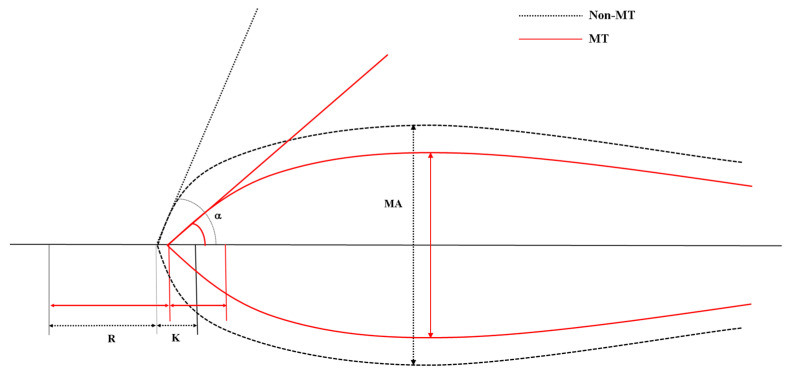
Graphical illustration of thromboelastographic recording and parameters of the non-MT (black dotted line) and MT (red solid line) groups. MT, massive transfusion; R, reaction time; K, kinetic time; MA, maximum amplitude.

The graphical illustration of the TEG parameters for median value of each group are shown in Figure 2. The K, MA, and alpha angle values among the TEG values were found to be discriminative, with AUCs > 0.750. The optimal cutoff values for predicting the need for MT using TEG were K > 1.5 min, alpha angle < 60 degrees, and MA < 63 mm.

### 3.3. Predicting Factors Associated with the Need for Massive Transfusion

We performed multivariate analysis to identify independent variables associated with the need for MT, including initial mental status, lactate level, shock index > 0.9, and groups defined using TEG values (K > 1.5 min, alpha angle < 60 degrees, and MA < 63 mm), as shown in Table 4. In the multivariate regression analysis, we found three clinical factors to be independently associated with the requirement of MT: lactate (OR 1.674, 95% CI 1.218–2.300; *p* = 0.001), initial shock index > 0.9 (OR 4.638, 95% CI 1.784–12.056; *p* = 0.002), and alpha angle < 60 degrees (OR 7.769, 95% CI 2.736–22.062; *p* < 0.001).

### 3.4. Performance Parameters for TEG values and Shock Index to Predict the Need for Massive Transfusion

Table 5 presents performance parameters for TEG values and shock index to predict the need for massive transfusions in study populations. The sensitivity was highest at 88.2% for K > 1.5, followed by MA < 63 and angle < 60. Otherwise, angle < 60 had the highest specificity of 72.0%, followed by MA < 63 and K > 1.5. All variables demonstrated excellent negative predictive values exceeding 90%. Angle < 60 demonstrated superior sensitivity, positive predictive value, and negative predictive value in comparison to shock index > 0.9.

## 4. Discussion

In this study, we identified significant differences in TEG values between the MT group and the non-MT group. Along with objective parameters such as the initial shock index and lactate levels, a TEG value with an alpha angle < 60 degrees was found to be independently associated with the need for MT. Alpha angle < 60 degrees indicated the need for MT with 73.5% sensitivity, 72.0% specificity, and 92.3% negative predictive value. These findings suggest that point-of-care TEG testing could be a helpful tool for identifying patients with primary PPH who require MT upon ED admission.

The prevalence of MT in ED patients with primary PPH was approximately 20% in our study. Previous studies in high-income countries using a nationwide cohort have reported that the annual incidence of MT in PPH ranged from 23 to 91 per 100,000 births [20,21]. However, the prevalence of patients with PPH visiting the ED was significantly higher. According to Kong et al., who evaluated the delta neutrophil index to predict MT in PPH in the ED, 21.6% of patients required MT, which is consistent with our study [22]. As most patients with PPH were referred from outside clinics or hospitals because of uncontrollable bleeding, this may explain the higher prevalence of MT in the ED. Given the higher incidence of MT in the ED and the unfavorable outcomes that result from delayed intervention [23], it is crucial to improve patient outcomes by promptly identifying those at a higher risk.

Recent treatment guidelines emphasize the importance of identifying and managing coagulopathy, as well as providing resuscitation [9]. Coagulopathies can worsen bleeding and contribute to the occurrence of severe bleeding. However, early coagulopathy is uncommon during the initial phase of PPH, except in rare cases such as amniotic fluid embolism and placental abruption [16,24]. The prevalence of coagulopathy in PPH is estimated to be approximately 3%, and this rate increases with the severity of the bleeding [16]. As previously discussed, patients referred to the ED with PPH may have a more advanced stage of bleeding. Therefore, it is likely that there will be more changes in their coagulation status.

Nevertheless, the hemostatic alterations observed in PPH exhibit distinct characteristics when compared to bleeding in other settings, such as trauma [25]. In pregnant women, it has been observed that conventional coagulation tests including aPTT and PT, generally yield normal results despite significant blood loss of up to 5000 mL [26,27,28]. In other critical illnesses, such as sepsis, a concern exists regarding the potential inaccuracy of conventional coagulation tests in reflecting the intricate nature of coagulation disturbances that occur in vivo [29]. The use of TEG might be beneficial in accurately evaluating the coagulation status, given the dynamic clinical presentation of PPH.

This study found significant differences in the TEG values of K, alpha angle, and MA, which are indicators of fibrinogen activity, between the MT group and the non-MT group. In cases of severe bleeding, such as PPH, the fibrinogen concentration in the plasma reaches critically low levels prior to the concentrations of coagulation factors [30,31]. It suggests that using TEG to promptly identify hypofibrinogenemia may have potential benefits in treating coagulopathy in patients with PPH, aligning with the results of prior research [32,33].

However, there could be a concern about the feasibility: if the hospital is not available to use the viscoelastic testing in our study, our results would not be applicable to use in practice. We have shown that other coagulation parameters, such as platelets, fibrinogen, FDP, and D-dimer, were significantly different between the groups. Additionally, the pregnancy-modified ISTH score showed a significant difference. Therefore, a conventional coagulation test along with a DIC score might be enough to help a physician manage PPH. However, a previous study found that TEG could identify overt DIC with 95.2% sensitivity and an AUG of 0.957 [34]. Given its short turnaround time at bedside, TEG could allow prompt recognition and management of coagulopathy in pregnancy, along with other conventional coagulation parameters [35].

In this study, we found that a TEG value of alpha angle < 60 degrees was an independent variable associated with the requirement for MT. Given the significant risk of maternal mortality in PPH, it is essential to conduct early risk assessment and expedite resuscitation to improve the overall prognosis [36]. In a previous study, we provided evidence that combining the initial shock index with lactate levels is an effective method for identifying patients who require MT [18]. One notable advantage of these characteristics is their accessibility and ease of application for patients upon ED admission. Due to its nature, the TEG point-of-care test can be used in conjunction with shock index and lactate measurements upon patient admission. A rapid risk assessment tool could be beneficial for PPH. Further study is necessary to adopt a multidisciplinary approach that incorporates vital signs, hemodynamic changes, and coagulation status to determine the need for MT in PPH.

There are several limitations in our study. First of all, we conducted the study including patients who visited the ED after delivery at a single center. As a result, generalizing our findings to patients in obstetric clinics, hospital delivery units, or other healthcare facilities would not be appropriate. Additionally, the study design used was retrospective, which could potentially introduce biases and confounding variables that may impact the validity of our findings. Second, the implementation of the MT protocol occurred during the study period. Consequently, there may have been variations in clinical practices for patients with primary PPH throughout the study. Third, we only used the initial value of TEG to determine the need for MT. Given the dynamic nature of PPH’s clinical manifestations, it would be more beneficial to have data on the serial follow-up of TEG values. Additionally, it is possible that the optimal timing for implementing TEG may not coincide with the moment of admission. Further study is needed to clarify future investigations.

## 5. Conclusions

We found that that point-of-care assessment of TEG has the potential to be an effective tool for the early prediction of the requirement for MT in patients who have PPH. The measurement of TEG upon admission, in conjunction with other objective parameters like lactate and shock index, can be of assistance in immediately stratifying patients according to their risk and directing early interventions like transfusion strategies. A future study with protocolized treatment with TEG will be needed to elucidate the role of TEG in the management of PPH.

## Figures and Tables

**Table 1:** Comparisons of baseline and clinical characteristics in emergency department patients with primary postpartum hemorrhage, based on the need for massive transfusion.

Variables	MT Group (n = 34)	Non-MT Group (n = 150)	*p* Value
Age, years	34.1 ± 4.2	32.9 ± 4.1	0.127
Parity			0.085
Primipara	16 (47.1)	95 (63.3)	
Multipara	18 (52.9)	55 (36.7)	
Delivery type			0.442
Vaginal delivery	23 (67.6)	90 (60.0)	
Cesarean section	11 (32.4)	60 (40.0)	
Initial mental status			<0.001
Alert	28 (82.4)	149 (99.3)	
Verbal	4 (11.8)	1 (0.7)	
Painful	1 (2.9)	0 (0.0)	
Unresponsive	1 (2.9)	0 (0.0)	
Initial vital signs			
Systolic blood pressure, mmHg	103.0 (75.0–121.0)	115.0 (105.0–129.0)	0.001
Diastolic blood pressure, mmHg	63.0 (50.0–72.0)	73.0 (62.0–84.0)	0.003
Heart rate, beats/min	114.0 (100.0–124.0)	90.0 (80.0–101.0)	<0.001
Body temperature, °C	37.5 (37.0–38.0)	37.4 (36.9–38.0)	0.655
Shock index	1.1 (0.8–1.5)	0.8 (0.7–0.9)	<0.001

Values are expressed as the median (interquartile range) or as a number (%). MT, massive transfusion.

**Table 2:** Comparisons of initial laboratory findings, amount of blood transfusion, and clinical outcomes in emergency department patients with primary postpartum hemorrhage, based on the need for massive transfusion.

Variables	MT Group (n = 34)	Non-MT Group (n = 150)	*p* Value
Initial laboratory findings			
Lactate, mmol/L	2.8 (1.9–4.2)	2.1 (1.6–3.0)	0.006
Hemoglobin, g/dL	8.4 (6.7–10.3)	10.2 (9.1–11.7)	<0.001
Hematocrit, %	26.6 (20.9–31.2)	31.3 (27.7–35.2)	<0.001
Platelets, ×10^3^/µL	113.0 (95.0–143.0)	160.0 (133.0–201.0)	<0.001
Prothrombin time (INR)	1.4 (1.2–1.9)	1.1 (1.0–1.2)	<0.001
Fibrinogen, mg/dL	104.0 (50.0–167.0)	245.0 (169.0–325.0)	<0.001
FDP, µg/mL	115.0 (38.0–120.0)	48.0 (22.0–120.0)	0.13
D-dimer, µg/mL	35.2 (14.9–35.5)	13.4 (7.6–35.2)	0.018
Pregnancy-modified ISTH DIC score	51.0 (32.8–51.8)	26.0 (7.0–27.8)	<0.001
Amount of blood transfusion, units			
Packed red blood cells	12.0 (11.0–15.0)	3.0 (2.0–5.0)	<0.001
Fresh frozen plasma	11.0 (8.0–12.0)	2.0 (0.0–4.0)	<0.001
Platelet concentrates	10.0 (8.0–16.0)	0.0 (0.0–0.0)	<0.001
Clinical outcomes			
Embolization	30 (88.2)	61 (40.7)	<0.001
Hysterectomy	2 (5.9)	0 (0.0)	0.033
Length of hospital stay, days	4.0 (2.0–5.0)	2.0 (1.0–3.0)	<0.001
ICU admission	2 (5.9)	5 (3.3)	0.615
In-hospital mortality	1 (2.9)	0 (0.0)	0.185

Values are expressed as the median (interquartile range) or as a number (%). MT, massive transfusion; INR, international normalized ratio; FDP, fibrin degradation product; ICU, intensive care unit.

**Table 3:** Comparisons of thromboelastographic parameters based on the need for massive transfusion in emergency department patients with primary postpartum hemorrhage.

Variables	MT Group (n = 34)	Non-MT Group (n = 150)	AUC	*p* Value
R, min	4.3 (3.2–6.4)	3.8 (3.2–4.4)	0.621	0.028
K, min	3.9 (2.2–6.4)	1.5 (1.2–2.3)	0.813	<0.001
Alpha angle, degrees	47.5 (33.1–61.1)	68.0 (58.4–73.1)	0.794	<0.001
MA, mm	48.9 (34.7–59.3)	64.1 (56.1–68.6)	0.801	<0.001
LY30, %	0.0 (0.0–1.0)	0.0 (0.0–0.3)	0.527	0.58

Values are expressed as the median (interquartile range) or as a number (%). MT, massive transfusion; AUC, area under the curve; R, reaction time; K, kinetic time; MA, maximum amplitude; LY30, lysis after 30 min.

**Table 4:** Multivariate logistic regression analysis identifying the independent factors associated with the need for massive transfusion in emergency department patients with primary postpartum hemorrhage.

Variables	Adjusted Odds Ratio	95% Confidence Interval	*p* Value
Lactate, mmol/L	1.674	1.218–2.300	0.001
Shock index > 0.9	4.638	1.784–12.056	0.002
Alpha angle < 60 degrees	7.769	2.736–22.062	<0.001

**Table 5:** Performance parameters for TEG values and shock index to predict the need for Massive Transfusions in study population.

Variables	Sensitivity (%)	Specificity (%)	PPV (%)	NPV (%)
K > 1.5	88.2 (72.6–96.7)	52.0 (43.7–60.2)	29.4 (25.3–33.9)	95.1 (88.5–98.0)
Angle < 60	73.5 (55.6–87.1)	72.0 (64.1–79.0)	37.3 (30.0–45.2)	92.3 (87.2–95.5)
MA < 63	85.3 (68.9–95.1)	54.7 (46.3–62.8)	29.9 (25.4–34.8)	94.3 (87.8–97.4)
SI > 0.9	67.6 (49.5–82.6)	72.7 (64.8–79.6)	35.9 (28.3–44.3)	90.8 (85.8–94.2)

## Data Availability

Data are contained within the article.
